# Expression, purification and structural analysis of functional GABA transporter 1 using the baculovirus expression system

**DOI:** 10.3762/bjoc.13.88

**Published:** 2017-05-11

**Authors:** Jing Hu, Chris Weise, Christoph Böttcher, Hua Fan, Jian Yin

**Affiliations:** 1 Wuxi School of Medicine, Key Laboratory of Carbohydrate Chemistry and Biotechnology, Ministry of Education, Jiangnan University, Lihu Avenue 1800, 214122, Wuxi, China; 2 Institut für Biochemie und Molekularbiologie, Campus Benjamin Franklin, Charité Universitätsmedizin Berlin, Arnimallee 22, Berlin D-14195, Germany; 3 Forschungszentrum für Elektronenmikroskopie, Institut für Chemie und Biochemie, Freie Universität Berlin, Fabeckstr. 36a, Berlin D-14195, Germany; 4 Institut für Chemie und Biochemie - Biochemie, Freie Universität Berlin, Thielallee 63, Berlin D-14195, Germany; 5 Institut für Laboratoriumsmedizin, Klinische Chemie und Pathobiochemie, Charité Universitätsmedizin Berlin, Berlin D-13353, Germany; 6 Key Laboratory of Carbohydrate Chemistry and Biotechnology, Ministry of Education, School of Biotechnology, Jiangnan University, Lihu Avenue1800, Wuxi 214122, China

**Keywords:** baculovirus expression system, chromatography, γ-aminobutyric acid (GABA), GABA transporter 1 (GAT1)

## Abstract

The γ-aminobutyric acid (GABA) transporter 1 (GAT1) belongs to a family of Na^+^ and Cl^−^-coupled transport proteins and possesses 12 putative transmembrane domains. To perform structural analyses of the GAT1 protein, the GAT1/green fluorescent protein (GFP) fusion protein was functionally expressed in insect *Sf*9 cells by the BAC-TO-BAC^®^ baculovirus expression system. A two-step procedure to purify the GAT1/GFP fusion protein from insect *Sf*9 cells has been established and involves immunoaffinity chromatography using self-prepared anti-GFP antibodies and size-exclusion fast protein liquid chromatography (SE-FPLC). A yield of 200–300 μg of the GAT1/GFP protein could be purified from 400–600 mL of infected *Sf*9 cells. The purified protein was analyzed by transmission electron microscopy (TEM), which revealed that the GAT1/GFP fusion protein was isolated in its monomeric form.

## Introduction

γ-Aminobutyric acid (GABA) is the most abundant inhibitory neurotransmitter in the central nervous system (CNS) of vertebrate species. GABAergic neurotransmission is efficiently terminated through the quick removal of GABA from the synaptic cleft by GABA transporters (GATs). The activities of GATs are important for controlling the concentration and dwell time of GABA in the synaptic cleft and tightly regulating the synaptic inhibition of the GABA receptor [1]. GATs, which are located in the plasma membranes of neurons and glia cells, belong to the solute carrier 6 (SLC6) family in mammals, which is subdivided into four groups based on sequence composition: GATs, GABA, osmolyte and creatine transporters, neurotransmitter amino acid, monoamine and nutrient amino acid/orphan transporters [2–3]. Four subtypes of GATs (GAT1–4) have been identified thus far [4].

GAT1, the first neurotransmitter transporter to be cloned, is abundantly but restrictively expressed throughout rat, mouse, and human CNSs [4–8]. The transmembrane (TM) topology of GAT1 demonstrates that this single polypeptide contains twelve TM domains connected by hydrophilic loops with the amino and carboxy-termini residing in the cytoplasm [9–10]. Additionally, the GAT1 protein contains three conserved N-glycosylation sites [9]. The role of N-linked glycans in the GABA uptake activity of GAT1 has been extensively clarified [11–12]. The topological model of GAT1 is in agreement with the high-resolution crystal structure of LeuT_Aa_, a homologue of the SLC/neurotransmitter sodium symporter (NSS) transporters from the bacterium *Aquifexaeolicus* [13]. The LeuT_Aa_ structure has provided a good model for the study of GAT1 and other SLC6 members [3]. The activities of GATs are driven by electrochemical gradients of Na^+^ and Cl^−^ ions [14], and the binding pocket for the substrate and two sodium ions has been clearly documented in the crystal structure of LeuT_Aa_. The chloride dependence has also been elucidated by identifying crucial structural elements for chloride binding [15–16]. In addition, a similar core architecture was observed based on several crystal structures of sodium symporters from different families; this observation strongly supports an alternating access mechanism as a common transport mechanism for the GAT1 protein [13,17–20].

Since the GABAergic system has been implicated in many nervous system diseases [21–24], the regulation of GABA activity is of considerable medical interest [25–26] and the predominant GABAergic nerve ending protein, GAT1, is a potential drug target. The exact three-dimensional structure of GAT1 protein could provide more information for pharmaceutical research.

The structural analysis of most membrane proteins is challenging since significant protein yields are required and because eukaryotic membrane proteins should be in a native and functional conformation during expression and purification. The natural abundance of most membrane proteins is typically not high enough for the isolation of sufficient quantities for functional and structural studies. In this work, the baculovirus expression system was selected for GAT1 protein expression. Mass spectrometry, gel electrophoresis and GABA uptake assays were employed to characterize the product during expression and purification. In our work, the GAT1 protein with a green fluorescent protein (GFP) tag at its C-terminus, which does not affect the relevant functions of GAT1 [27], could be purified in a functional form. In addition, the GFP tag has several advantages for further work. For example, it provides a powerful means for affinity purification with specific anti-GFP antibodies that have already been produced, generates green fluorescence for the efficient observation of the expression of the protein of interest, and has a known crystal structure [28–29] for future structural studies.

## Results and Discussion

### Expression and characterization of the GAT1/GFP fusion protein in insect cells

#### Construction and analysis of the GAT1/GFP recombinant baculovirus

The purification of a protein of interest for structural studies requires an abundant source of the protein from heterologous overexpression. In our work, *Escherichia coli* was not a suitable system for the expression of the GAT1/GFP recombinant protein due to its twelve TM domains and N-glycosylation status. After 30 years of development, the baculovirus expression system has become a widely applied technology for producing recombinant proteins [30–31]. Since the production of adequate quantities of a homogenous protein is a rate-limiting step, in this study, we chose the baculovirus expression system for GAT1 protein expression instead of the mammalian cell expression system.

**Figure 1:**
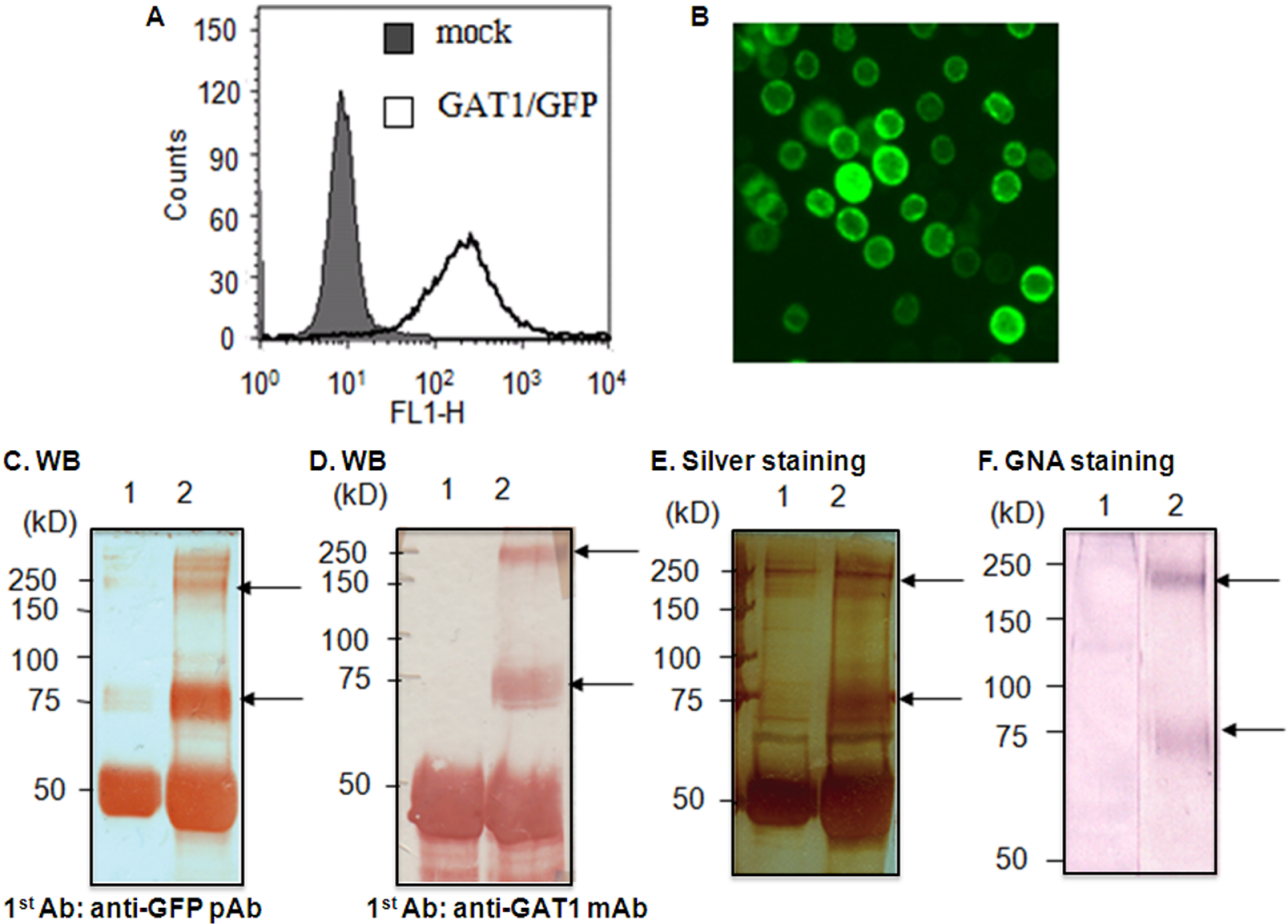
Expression of GFP-tagged GAT1 in infected insect cells. (A) Flow cytometry analysis of GAT1/GFP in *Sf*9 cells. (B) Fluorescence microscopy of infected *Sf*9 cells. The GFP fluorescence in the GAT1/GFP fusion protein was detected. The solubilized proteins of infected *Sf*9 cells were subjected to immunoprecipitation with anti-GFP mAb IgG and then analyzed by SDS-PAGE (7.5%) and immunoblotting. (C) and (D) Western blotting analysis of GAT1/GFP from uninfected and infected *Sf*9 cells with anti-GFP pAb or anti-GAT1 pAb. (E) Silver staining of GAT1/GFP from infected *Sf*9 cells. (F) GNA staining of GAT1/GFP from infected *Sf*9 cells. The main bands corresponding to the GAT1/GFP fusion protein are indicated with arrows; *Sf*9 cells (lane 1), GAT1/GFP infected *Sf*9 cells (lane 2).

The GAT1/GFP recombinant cDNA was cloned into the pFastBac1 vector (Figure S1A, Supporting Information File 1) and prepared in bacmids (Figure S1B, Supporting Information File 1) for the baculovirus expression system. GAT1/GFP recombinant proteins were expressed in *Sf*9 cells by viral infection for 72 h. The expression of the GAT1/GFP protein was further characterized. After 72 h post-infection, *Sf*9 cells with green fluorescence were controlled by flow cytometry (Figure 1A) and analyzed by fluorescence microscopy (Figure 1B). The expression of GAT1/GFP in insect cells was further determined by sodium dodecyl sulfate polyacrylamide gel electrophoresis SDS-PAGE, followed by Western blotting with either anti-GFP pAb (Figure 1C) or anti-GAT1 pAb (Figure 1D) and silver staining (Figure 1E) after immunoprecipitation with anti-GFP mAb (showing the band at approximately 50 kDa). In infected *Sf*9 cells, the GAT1/GFP-fusion protein shows two main bands in SDS-PAGE (7.5%) (indicated by arrows), and the monomeric form appears as a main band of approximately 75 kDa. This band can bind strongly only with *Galanthusnivalis* agglutinin (GNA, digoxigenin-conjugated lectin) (Figure 1F), indicating the predominance of the paucimannose structure in insect cells. In contrast, mammalian *N*-glycans have terminal sialic acid residues with more antennal diversity. The band at approximately 250 kDa corresponds to an oligomeric form or protein aggregate identified through further characterization.

**Figure 2:**
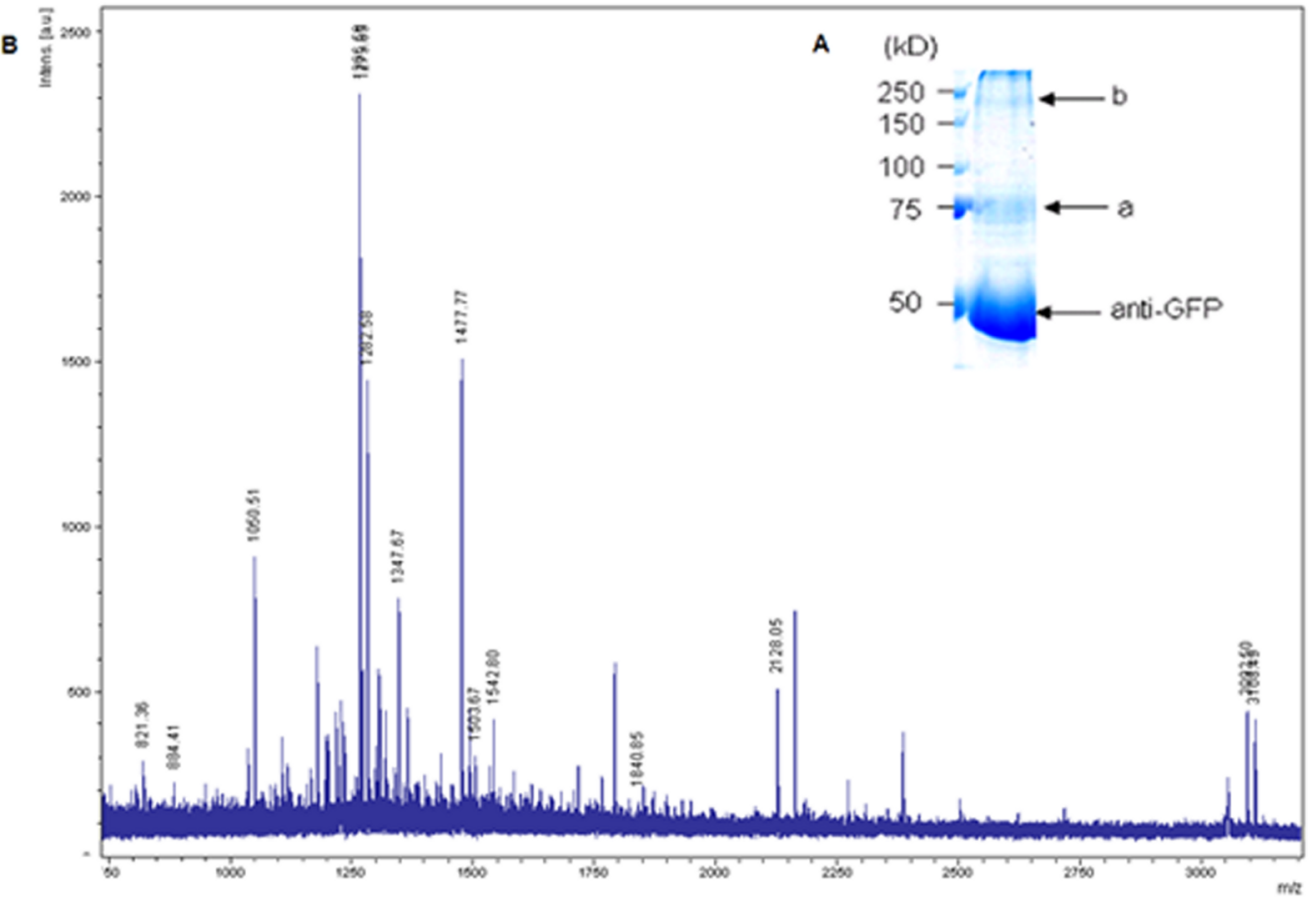
Protein fingerprinting. (A) Coomassie Blue staining of the GAT1/GFP protein from infected *Sf*9 cells after immunoprecipitation. Bands a and b (indicated by arrows) were extracted for MALDI–TOF analysis. (B) Mass spectrum of the trypsin digest of band a. The labeled peaks were assigned to either GAT or GFP.

The two main protein bands after immunoprecipitation and SDS-PAGE with Coomassie Blue staining (a and b in Figure 2A) were extracted for protein fingerprinting analysis. The extracted proteins were processed by trypsin treatment, and tryptic peptides were analyzed either directly by matrix-assisted laser desorption/ionization-time-of-flight mass spectrometry (MALDI–TOF MS) or guanidinated and then analyzed by MALDI. The labeled peptide peaks (Figure 2B) in the mass spectrum were identified as either GAT or GFP by matching the peptides from primary sequence databases with Mascot (http://www.matrixscience.com/).

The biological function of recombinant proteins of mammalian origin expressed in insect cells may be altered by different *N*-glycan status. We observed that the terminal sialic acid residues are essential for the GABA uptake activity of GAT1 because they affect the ionic affinity for Na^+^ and the conformational change of the GAT1 protein during its uptake process [12]. After 72 h post-infection, the GABA uptake activity of *Sf*9 cells was measured to determine whether the GAT1/GFP-fusion protein was functionally expressed in the baculovirus expression system. The results showed that infected *Sf*9 cells (0.15 pmol/10^6^ cells) have only slightly higher GABA uptake activity than mock cells (0.1 pmol/10^6^ cells) (Figure S3, Supporting Information File 1). A previous work demonstrated that co-translational *N*-glycosylation but not the terminal trimming of *N*-glycans is involved in the regulation of the correct membrane glycoprotein folding since the inhibition of *N*-glycosylation processing by 1-deoxymannojirimycin (dMM) results in a mannose-rich type of *N*-glycan that does not affect either the protein stability or intracellular trafficking [11]. Therefore, the correct folding of GAT1/GFP protein in insect cells should not be affected by the lack of terminal trimming, including the sialylation of *N*-glycans. The low GABA uptake activity of GAT1/GFP fusion protein in insect cells should result from the terminal mannose structure on the *N*-glycans, which is consistent with previous findings [12]. Therefore, the GAT1/GFP protein produced in the baculovirus system can be suitable for further structural analysis with correct folding and more uniform, less complex *N*-glycans.

#### Purification of the GAT1/GFP fusion protein from insect cells by immunoaffinity chromatography and size-exclusion (SE) chromatography

A two-step purification procedure for the GAT1/GFP-fusion protein from *Sf*9 cells was established. The GAT1/GFP expressed in *Sf*9 cells was first isolated with mAb-GFP antibody-conjugated affinity chromatography. Subsequently, eluted fractions containing the GAT1/GFP protein were pooled and subjected to fast protein liquid chromatography based on SE (SE-FPLC) with a Superdex 200^TM^ column to obtain purified homogeneous GAT1/GFP fusion protein.

**Figure 3:**
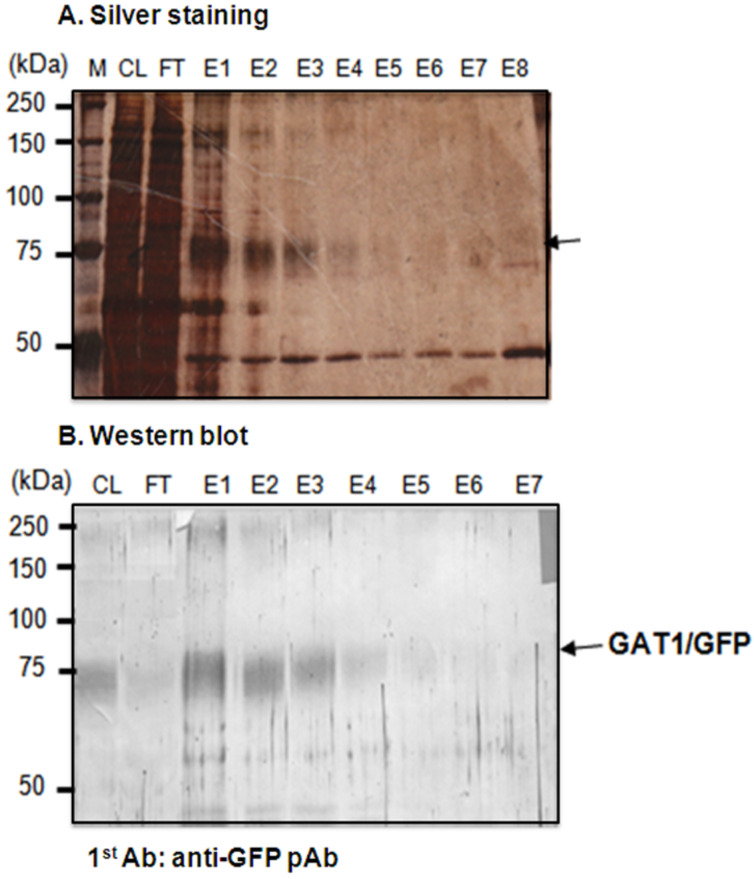
Isolation of the GAT1/GFP fusion protein from *Sf*9 cells by mAb-GFP-conjugated affinity column chromatography with analysis of the eluted fractions from the mAb-GFP-conjugated affinity column with 4 M MgCl_2_ (pH 6) by SDS-PAGE with silver staining and Western blotting. The arrow denotes the fragment of mAb-GFP. M: standard marker; CL: cell lysate; FT: flow-through; E1–E8: eluted fractions 1–8.

To isolate the GAT1/GFP protein from the monoclonal anti-GFP antibody (mAb-GFP)-conjugated affinity column, different elution buffers (with different pH values and ionic strengths) were tested to obtain an effective and appropriate elution condition for the GAT1/GFP protein without irreversibly denaturing or inactivating it. Since no easy and effective method to control the activity of GAT1 protein during purification exists, a near-neutral high-salt buffer containing 4 M MgCl_2_ (pH 6) (Figure 3) was selected to avoid the irreversible aggregation of GAT1, which may be caused by high pH values.

**Figure 4:**
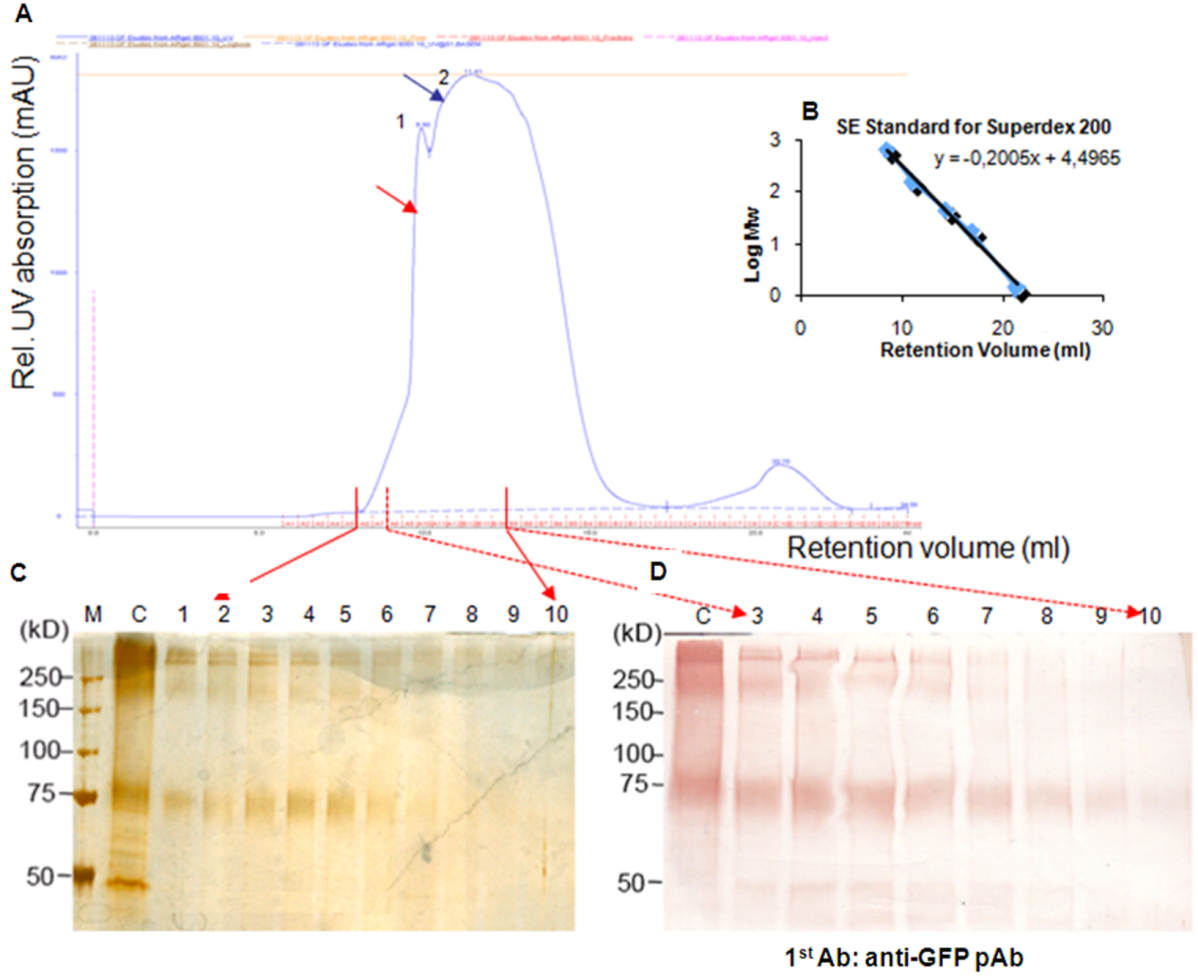
Purification of the GAT1/GFP fusion protein from *Sf*9 cells by SE-FPLC. (A) Elution profile of GAT1/GFP after SE-FPLC on a Superdex 200^TM^ column. (B) The standard linear regression curve of the Superdex 200^TM^ column was generated by plotting the log of the molecular masses of different calibration proteins against their elution volumes. (C), (D) Silver staining and Western blotting of the SDS-PAGE (7.5%) results of eluted fractions (from 8.8 to 11.8 mL) after SE-FPLC. M: standard marker; C: concentrated sample from immunoaffinity column; 1–10: SE-FPLC fractions from 8.8 to 10.8 mL.

**Figure 5:**
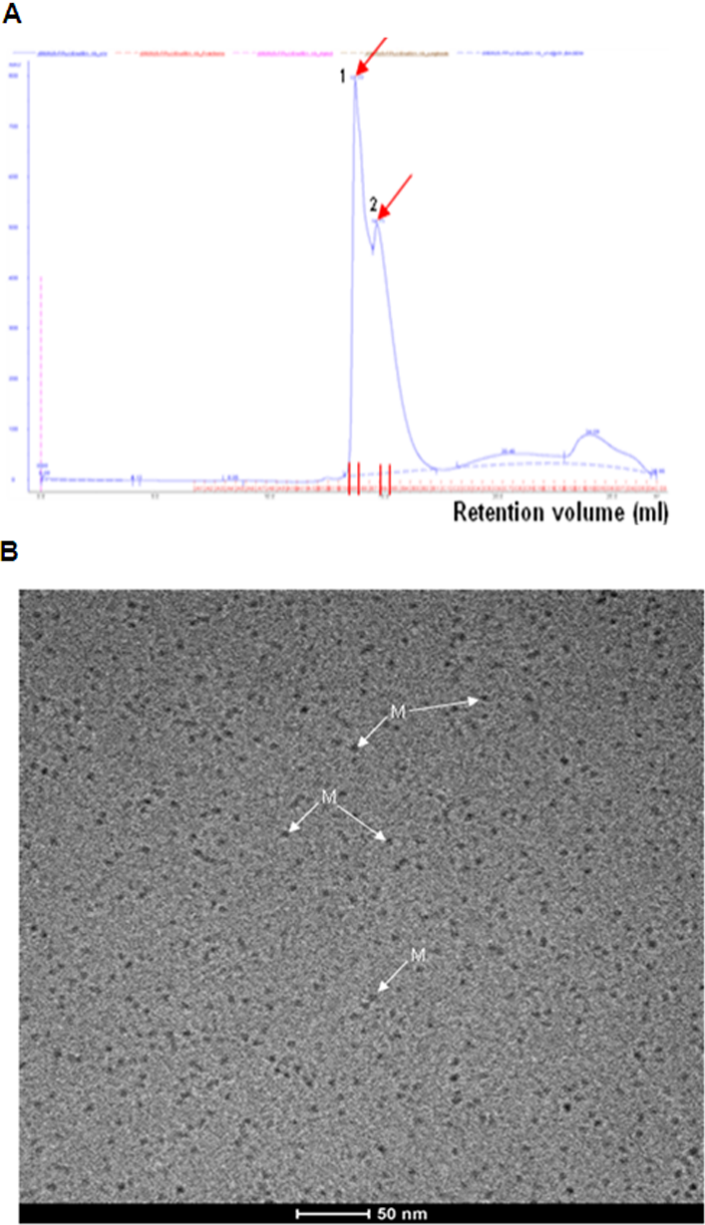
Characterization of the GAT1/GFP fusion protein after SE-FPLC. (A) Elution profile of GAT1/GFP protein after SE-FPLC on a Superdex 200^TM^ column. (B) Cryo-TEM image of fraction peak 2. Assumed monomeric GAT1/GFP fusion proteins (M, diameter = 5–6 nm) are indicated with white arrows. The control experiment revealed no significant population of spherical micelles.

A second purification step with SE-FPLC was performed to remove the eluted antibodies and other impurities from the eluates of the immunoaffinity column. The elution profile of the GAT1/GFP protein is shown in Figure 4A. Compared with the standard proteins (Figure 4B), two main peaks (1 and 2) appear, corresponding to *M*_r_ of 320 and 162 kDa, respectively. The fractions (300 μL per fraction) from 8.8 to 11.8 mL after SE-FPLC were further analyzed by SDS-PAGE (7.5%), followed with silver staining and Western blotting (Figure 4C and 4D). The results indicated that peak 1, which should correspond to a tetrameric GAT1/GFP with a molecular weight of 320 kDa, appeared at 8.8–10.6 mL (lanes 2–8). Peak 2 should correspond to a dimeric form with a molecular weight of 162 kDa, but despite its strong ultraviolet (UV) absorption, it contained very little GAT1/GFP protein according to the results of silver staining, Western blot and bicinchoninic acid (BCA) assay. To prevent the protein from forming oligomers during purification, different detergents were tested, and *n*-dodecyl-β-D-maltoside (DDM, 0.05%) was found to efficiently maintain the protein in its monomeric form (Figure 5A). The SE-FPLC elution profile is shown in Figure 5A. Peak 1, which appears at 13.5–14 mL, corresponds to a molecular weight of approximately 70 kDa, and the overlapping peak 2 (14.7 mL) corresponds to a molecular weight of 45 kDa, which was determined by comparison with the protein standards (Figure 4B).

#### Transmission electron microscopy (TEM) analysis

Cryogenic TEM (cryo-TEM) was employed to analyze both peaks from SE-FPLC to avoid the putative influences of sample drying and staining salts. The cryofixation by sample nitrifications is known to preserve the sample in the native state of the buffer environment and correspondingly the cryo-microscopy allows a direct visualization of the protein in the fully hydrated state. After a BCA control, the fraction of peak 2 containing the larger amount of protein was characterized by cryo-TEM and showed a very monodisperse distribution of particles (Figure 5B) with a diameter in the 5–6 nm range, which might correspond to protein monomers. The peak 1 fraction contains only a low amount of the GAT1/GFP protein that exits partly in an aggregated form (Figure S4, Supporting Information File 1).

Some noticeable small particles with high contrast were observed. The radius of DDM micelles has been reported to be approximately 2.6–3.5 nm [32], however, the observed high contrast is not typical for detergent micelles. As it was not clear whether the particles could simply be attributed to spherical micelles control cryo-TEM experiments were performed. Using a Tris-buffered saline (TBS) solution containing 0.05% DDM (which is far above the critical micellar concentration (CMC) of DDM:0.009% or 0.18 mM), no significant population of micelles could be found. These results suggest that the observed particles most likely correspond to a monodisperse preparation of GAT1/GFP. Several studies have demonstrated that GAT1 expresses in an oligomeric formation in the plasma membrane. GAT1 dimers were expressed and examined as a distinct population of 9 nm freeze-fracture particles in the plasma membrane of *Xenopuslaevis oocytes* by freeze-fracture and electron microscopy [33]. The GAT1 protein monomer was determined to be the functional unit since each monomer functions independently [33–34]. A similar example is the bacteria homologue LeuT, which was also crystallized as a dimer [13], however, each monomer has its own binding pocket, indicating that the monomers are the functional units. Therefore, a GAT1/GFP fusion protein monomer could be suitable for further structural analysis. A yield of approximately 200–300 μg of GAT1/GFP protein in this fraction was obtained from 400–600 mL of infected *Sf*9 cells, quantified by the BCA assay.

## Conclusion

The aim of this project was to establish an expression and purification protocol for the production of high yields of GAT1/GFP fusion protein. In this work, the baculovirus expression system was used for the expression of the protein of interest. The full-length GAT1 protein is composed of twelve highly hydrophobic TM domains, which promote strong aggregation behavior of the protein, if it is isolated from the membrane. The presented protocol allows for the efficient production of the GAT1/GFP fusion protein in its monomeric form. We demonstrated that pure monomeric GAT1/GFP protein can be obtained with yields of approximately 200–300 μg from 400–600 mL of infected *Sf*9 cell culture. Moreover, considering the effect of *N*-linked glycans on the activity of the GAT1 protein, the glycol-engineered insect cells coupled with the baculovirus system may be further applied to produce a GAT1/GFP protein with complex, terminally sialylated *N*-glycans. Further structural analysis of the GAT1/GFP fusion protein is possible using crystallography, thereby improving our understanding of the three-dimensional structure of the GAT1 protein.

## Experimental

### Immunoprecipitation and Western blotting

The immunoprecipitation and Western blotting procedures were similar to those described previously [12]. Briefly, the GAT1/GFP protein was solubilized at 4 °C or on ice. After infection, insect cells were collected, washed once with phosphate-buffered saline (PBS), and resuspended in TBS buffer (50 mM Tris, pH 7.3, 150 mM NaCl). The suspended cells were sonicated at 4 °C for 15 min and cell debris was removed by centrifugation at 6,000*g* for 10 min at 4 °C. A turbid supernatant solution containing the cell membranes was obtained. After centrifugation at 100,000*g* at 4 °C for 30 min, the crude membrane fractions were solubilized in TBS containing 1% DDM and stirred for at least 4 h at 4 °C. The lysate was centrifuged at 18,000*g* at 4 °C for 1 h. Total protein concentrations of the supernatant were measured with BCA™ Protein Assay Kit (Thermo). Quantified aliquots of the supernatants were incubated with protein-G-Sepharose-bound anti-GFP IgG for 12 h at 4 °C. After intensive washing, the immunoprecipitates were eluted by boiling for 3 min in SDS sample buffer. The supernatant aliquots were divided in half and then subjected to SDS-PAGE; the separated proteins were transferred to a nitrocellulose membrane (Millipore) by Western blotting. One blot membrane was used for the immunostaining of the GAT1 protein with the anti-GAT1 or anti-GFP polyclonal antiserum. Subsequently, the blots were incubated with horseradish peroxidase-conjugated anti-rabbit antibody (IgG) (Dako Cytomation) and then visualized using amino ethylcarbazole (AEC) and substrate buffer (Calbiochem).

### Flow cytometry and fluorescence microscopy

*Sf*9 cells were observed after 3-day infection by flow cytometry and fluorescence microscopy to determine the cell surface expression of the GAT1/GFP fusion proteins.

### MALDI mass fingerprinting

Using MALDI–TOF MS, protein fragments with blocked N-termini or that are available in limited concentrations can be easily analyzed. The MALDI mass fingerprinting was performed by Dr. Chris Weise (Free University Berlin, Germany) with a Bruker-Biflex Reflex Mass spectrometer (Bruker Daltonics) in the reflector-mode with alpha-cyano-4-hydroxycinnamic acid as matrix. Ionization was enhanced with the 337 nm-ray of a nitrogen laser. The peptide masses were determined by calibration with the PAC peptide calibrant standard. All solutions and buffers used were prepared with sterile pure high-performance LC (HPLC)-grade water (Milli-Q^®^ Water filter apparatus, Millipore) in order to avoid contaminations. Evaluation and identification of the mass spectra were performed using the Internet search software Mascot [35].

### Purification of GAT1 and protein identification

#### Isolation of GAT1/GFP by the immunoaffinity column

The immunoaffinity column was prepared as followed: 200 µL Affi-gel 10 protein-A-sepharose (Amersham Pharmacia, Sweden) were loaded on a 10 mL disposable chromatography column and washed twice with 1 mL ice cold double-distilled (dd)H_2_O. Approximately 6–8 mg of purified antibody in 0.1 M MOPS buffer, pH 7.5, was added to the resin, and the columns were incubated overnight at 4 °C to allow covalent binding. Subsequently, unbound antibodies were collected as the flow-through, and the sepharose column was washed once with 1 mL of 1 M ethanolamine (pH 8.0). Blocking of the sepharose was performed with 3 mL of 1 M ethanolamine (pH 8.0) for 2 h at room temperature (rt). Finally, the antibody-coupled sepharose was washed three times with 15 mM sodium phosphate buffer (pH 8.0). Prior to subsequent use of the affinity columns, 1 mL of PBS with 0.02% NaN_3_ was added to the sepharose, which was then stored at 4 °C.

Cells were lysed in solubilization buffer (10 mM Tris, pH 7.8, 150 mM NaCl, 1 mM CaCl_2_, 2% DDM) containing 1 mM dithiothreitol (DTT) and protease inhibitor cocktail (1:500) (Merck). After a 4 × 30 s sonication step, the cells were incubated overnight at 4 °C with agitation. The solubilized protein was fractionated by 45 min of centrifugation at 18,000 rpm.

The cell lysate was added directly to the immunoaffinity chromatography resin. Proteins were coupled on the column by overnight agitation at 4 °C. Unbound proteins were collected as flow-through and the resin was washed three times with radio immunoprecipitation assay buffer (RIPA) and twice with prewash. The elution of the GAT1/GFP was achieved with 8 × 250 µL of elution buffer (50 mM diethylamine, pH 11.4). The eluates were neutralized immediately by adding 100 µL of 0.5 M NaH_2_PO_4_ to each tube in which the eluates were collected.

#### FPLC based on size exclusion (SE-FPLC)

The immunopurified GAT1/GFP isolated from the immunoaffinity column was concentrated to 250–300 μL with a Vivaspin column (Vivasciences) at 4 °C and 3,000*g*. The concentrate was further purified by SE-FPLC on a Superdex 200 column (GE Healthcare) that had been equilibrated with equilibrium buffer (10 mM Tris, pH 7.8, 150 mM NaCl, and 0.05% DDM). Elution of the protein was performed with equilibrium buffer at a flow rate of 0.3 mL/min. The molecular weight of the obtained protein was determined based on the elution profile of proteins standards obtained under the same buffer conditions.

### TEM analysis

#### Negative staining preparation

Sample droplets (5 μL) of the sample were placed onto hydrophilized (glow discharged for 60 s at 8 W in a BALTEC MED 020 device (Baltec, Liechtenstein) carbon-covered microscopical copper grids (400 mesh), and the supernatant fluid was removed with a filter paper to create an ultrathin layer of the sample. A droplet of contrasting material (1% uranyl acetate, 2% phosphotungstic acid or 2% ammonium molybdate in the presence of 0.1% trehalose) was added, blotted again and air-dried. Imaging was performed using a Tecnai F20 FEG (FEI Company, Oregon) at an accelerating voltage of 160 kV under low-dose conditions. Micrographs were recorded according to the low-dose protocol of the microscope at a primary magnification of 62,000×. The defocus value was chosen to correspond to the first zero of the contrast transfer function (CTF) at ≈15 Å.

#### Cryo-TEM

Similarly as described previously [36], vitrified samples were transferred into a Tecnai F20 FEG using a Gatan cryo-holder and -stage (Model 626). Samples were constantly cooled by LN2 during imaging to maintain a sample temperature of *T* = 93 K. Imaging was performed at an accelerating voltage of 160 kV with a defocus value of 600 nm, which corresponds to the first zero of the CTF at 13 Å (Cs = 2.0 mm). Micrographs were recorded according to the low-dose protocol of the microscope at a primary magnification of 62, 000×.

## Supporting Information

File 1 Additional information.
