# Supplementary material for: Expression, purification and structural analysis of functional GABA transporter 1 using the baculovirus expression system
Source: Beilstein J Org Chem. 2017 May 11;13:874–82. doi: 10.3762/bjoc.13.88 (PMC5433199; doi:10.3762/bjoc.13.88)
Supplement: File 1 — Additional information. [file Beilstein_J_Org_Chem-13-874-s001.pdf]

Supporting Information

for

Expression, purification and structural analysis of functional GABA transporter 1 using the baculovirus expression system

Jing Hu[1,2*], Chris Weise[3], Christoph Böttcher[4], Hua Fan[5] and Jian Yin[6*]

Address: [1]Wuxi School of Medicine, Key Laboratory of Carbohydrate Chemistry and Biotechnology, Ministry of Education, Jiangnan University, Lihu Avenue 1800, 214122, Wuxi, China, [2]Institut für Biochemie und Molekularbiologie, Campus Benjamin Franklin, Charité Universitätsmedizin Berlin, Arnimallee 22, Berlin D-14195, Germany, [3]Forschungszentrum für Elektronenmikroskopie, Institut für Chemie und Biochemie, Freie Universität Berlin, Fabeckstr. 36a, Berlin D-14195, Germany, [4]Institut für Chemie und Biochemie - Biochemie, Freie Universität Berlin, Thielallee 63, Berlin D-14195, Germany, [5]Institut für Laboratoriumsmedizin, Klinische Chemie und Pathobiochemie, Charité Universitätsmedizin Berlin, Berlin D-13353, Germany and [6]Key Laboratory of Carbohydrate Chemistry and Biotechnology, Ministry of Education, School of Biotechnology, Jiangnan University, Lihu Avenue1800, Wuxi 214122, China

Email: Jing Hu - hujing@jiangnan.edu.cn; Jina Yin - jianyin@jiangnan.edu.cn

*Corresponding author

Construction and analysis of GAT1/GFP recombinant baculovirus

The GAT1/GFP recombinant cDNA was cloned in the pFastBac1 vector (Figure S1A). After transfection of these bacmids, the primary recombinant baculovirus stock was harvested and used for viral amplification. The Bacmid DNA content of the virus was purified with the “Zymoclean DNA clean and concentration kit” and used as template for PCR with the M13 amplification prime rstobe further verified. Bacmid transposed with GAT1/GFP in pFastBac1 vector contains about 5,000 bp. As shown on agarose gel electrophoresis (Figure S1B), the GAT1/GFP DNA constuct was stable and correct for use in expression of the fusion protein in insect cells.

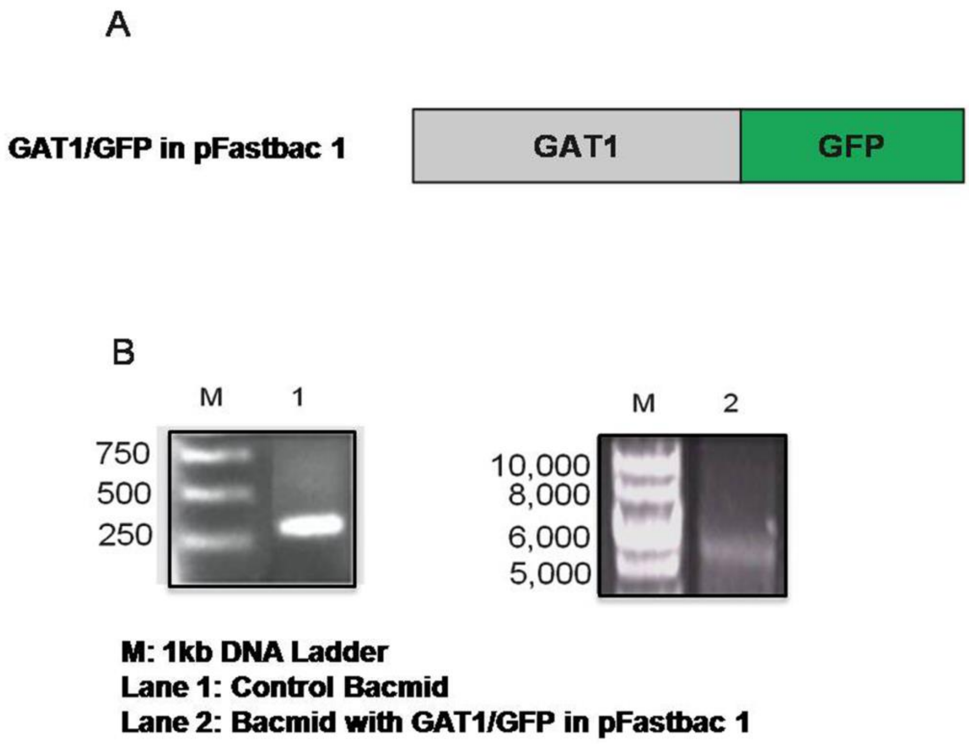

Figure S1: Construction of GAT1/GFP recombinant baculovirus. (A) Schematic representation of the GAT1/GFP fusion protein constructs cloned into the pFastBac1 vector. (B) PCR-analysis of recombinant baculovirus. Recombinant baculoviruses for the construct were verified by PCR for the presence of the GAT1/GFP cDNA with M13 amplification primers. Lane 1: Control Bacmid with around 300 kb; Lane 2: Bacmid with GAT1/GFP in pFastbac1 vector containing 5,000 bp.

Protein fingerprinting

After comparison between mass spectra for bands a and b, both spectra contain the same signals for GAT and GFP for selected peptides, *m*/*z* 1266/1282 for GFP (Figure S2A) and *m*/*z* 2128 for GAT (Figure S2B). The results identified that band b contains the GAT1/GFP protein in an oligomeric or aggregated form.

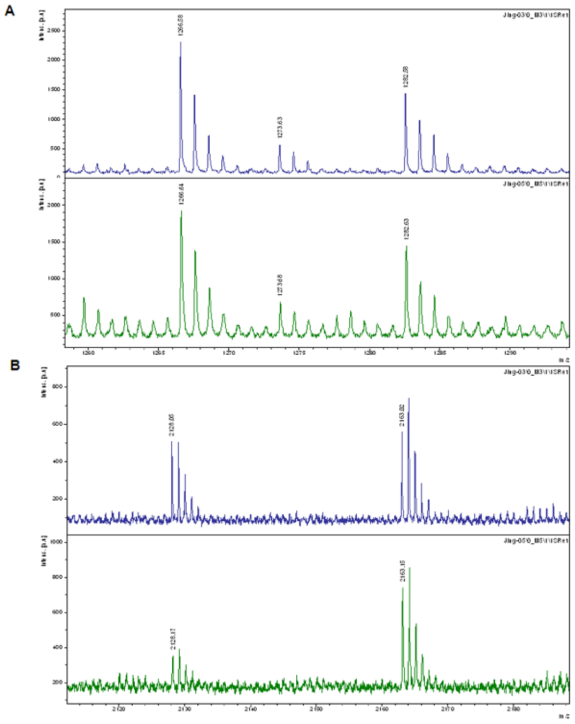

Figure S2: Protein finger printing. (A) The typical peaks (*m*/*z* 1266/1282) for GFP. (B) The typical peak (*m*/*z* 2128) for GAT. Blue spectrum: from band a; green spectrum: from band b.

GABA uptake activity of GAT1/GFP in insect cells

It has showed that infected *Sf*9 cells (0.15 pmol/10^6 cells) had only a little higher GABA uptake activity than that of mock cells (0.1 pmol/10^6 cells) (Figure S3). The terminal mannose structure on *N*-glycans of the GAT1/GFP fusion protein in insect cells may result in the decrease the GABA uptake activity.

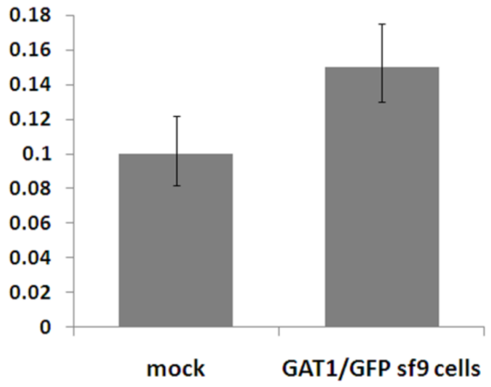

Figure S3: Functional determination of GFP-tagged GAT1 in *Sf*9 cells by the GABA uptake assay. The GABA uptake activities were normalized to the total cell number. The values represent the mean ± SD of at least three separate experiments.

Transmission electron microscopy (TEM) analysis

The fraction of peak 1 was analyzed by negative staining preparation with TEM. The electron micrograph shows a mixture of different forms, namely small particles of 5 nm diameter as before but also a considerable fraction of fiber-like aggregates probably due to the preparation conditions (Figure S4).

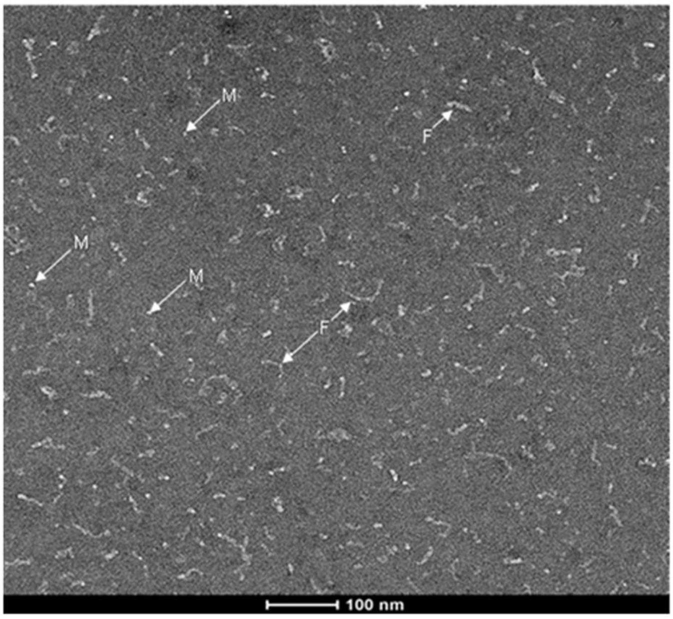

Figure S4: Electron micrograph (negative staining preparation with 1% uranyl acetate) of fraction peak 1. M: Assumed monomers of the GAT1/GFP fusion protein (M, diameter = 5–6 nm) and fiber-like aggregates are indicated.

Experimental

Construct preparation and expression of GAT1/GFP recombinant baculovirus

The BAC-TO-BAC^{TM}- Baculovirus expression system was utilized in this work for the expression of recombinant protein in *Sf*9 insect cells. GAT1/GFP recombinant Bacmid DNAs were constructed as following: The DNA construct GAT1/GFP fusion protein was constructed as described previously [1]. One µg plasmid-DNA was added to 100 µL DH10BAC^{TM} competent cells and incubated on ice for 30 min. After heat shocking at 42 °C for 45 s, the cells were incubated on ice for 2 min. SOC medium (900 µL) was added and mixed briefly. The cells were incubated for 4 h at 37 °C in a thermo-mixer with mixing at 500 rpm. 100 µL aliquots were plated on solid LB agar selection medium containing the antibiotics kanamycin, tetracycline and the substrates bluo-gal and IPTG. Positive clones remained white and were selected with blue-white selection after the plates were incubated for 48 h at 37 °C. Overnight cultures of the white clones were prepared in LB medium containing kanamycin and tetracycline and the recombinant Bacmids were purified with the Plasmid purification kit (Qiagen).

*Sf*9 cells were infected in 100 mL suspension cultures at a density of 2×10^6 cells/mL. For expression, cells were infected with the recombinant virus (generated, amplified and stored according to the baculoviruses preparation using the Cell FECTIN® Reagent) to final MOI rate 1, then incubated at 27 °C and shaking at 115 rpm for 3 d. Infected cells were harvested by centrifugation (2000 rpm for 5 min).

GAT1 activity assay

To determine the transport activity of GAT1 protein, [^{3}H]GABA uptake assays were performed as described previously [2]. Cells were washed three times with wash buffer (128 mM NaCl, 5.2 mM KCl, 2.1 mM $CaCl_2$, 2.9 mM $MgSO_4$, 5 mM dextrose and 10 mM Hepes, pH 7.4) and then incubated with 200 µL of wash buffer containing 3.7×10^4 Bq [^{3}H]GABA (Perkin Elmer), 10 µM cold GABA, 3.7×10^4 Bq [^{14}C]sucrose (Perkin Elmer) and 100 µM cold sucrose for 15 min at rt. The uptake was stopped by washing cells three times with cold wash buffer, followed by solubilization of the cells with 100 µL of 0.5% (w/v) SDS solution for 1 h at 4 °C. Aliquots were used for the measurement of the remaining [^{3}H]GABA and [^{14}C]sucrose. The protein concentration in the supernatant was determined using the bicinchoninic acid protein assay reagent

(Pierce). The GABA uptake activity was measured as pM μg protein^{-1} min^{-1}.

References

1. Cai, G.; Salonikidis, P. S.;Fei, J.; Schwarz, W.; Schulein, R.; Reutter, W.; Fan, H. *FEBS J.* **2005**, *272*, 1625-1638. doi:10.1111/j.1742-4658.2005.04595.x
2. Hu, J.; Fei, J.; Reutter, W.; Fan, H. *Glycobiology* **2011**, *21*, 329-339. doi:10.1093/glycob/cwq166
